# Best management in isolated right ventricular hypoplasia with septal defects in adults

**DOI:** 10.34172/jcvtr.2020.36

**Published:** 2020-08-27

**Authors:** Zahra Khajali, Maedeh Arabian, Maryam Aliramezany

**Affiliations:** ^1^Rajaie Cardiovascular Medical and Research Center, Iran University of Medical Sciences, Tehran, Iran; ^2^Cardiovascular Research Center, Institute of Basic and Clinical Physiology Sciences, Kerman University of Medical Sciences, Kerman, Iran

**Keywords:** Right Ventricular Hypoplasia, Adult Congenital Heart Disease, Cyanosis, Atrial Septal Defect

## Abstract

Hypoplastic right ventricle is a rare congenital disease usually associated with pulmonary atresia or tricuspid atresia. Isolated right ventricular hypoplasia is a rare anomaly without important valvular abnormalities. It is associated with inter atrial septal defects leading to the right-to-left shunting of blood. Patients with isolated right ventricular hypoplasia usually have different and variable courses. In some patients, it is recognized in the perinatal period and necessitates prompt intervention; nonetheless, there are some reports of this anomaly in old age with no significant symptoms. In this report, we describe the clinical data and management of 6 adult cases with isolated right ventricular hypoplasia treated medically or surgically based on the severity of the disease and symptoms and then offer an in-depth discussion regarding this rare anomaly.

## Introduction

Right ventricular hypoplasia, unassociated with severe pulmonary or tricuspid valvar malformations, is a primary congenital abnormality with the underdeveloped trabeculated sinus of the ventricle. In right ventricular hypoplasia, a patent foramen ovale or an atrial septal defect can serve as an escape valve.^1^ The clinical manifestations are usually nonspecific and vary related to the degree of hypoplasia and right ventricle compliance as well as the degree of right-to-left shunting via the atrial septal defect or the patent foramen ovale. Physical examinations are often normal.^2^ The diagnosis and evaluation of the severity of the syndrome are routinely performed by echocardiography, cardiac magnetic resonance imaging, and hemodynamic evaluation.^3^ The mild hypoplasia of the right ventricle can be corrected by atrial septal defect closure; nevertheless, for subjects with severe hypoplasia, the Glenn shunt, one and half ventricle repair, or even the Fontan surgery should be chosen.^4^

This report describes the history, para-clinical data, and management of 6 patients with isolated RVH treated medically or surgically based on their symptoms.

## Case 1

**Figure 1:**
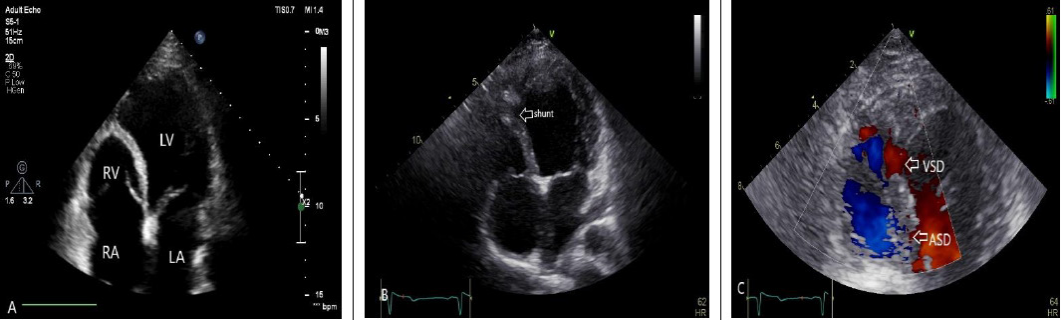
Two dimensional and color Doppler echocardiography showed: A) small and hypo-plastic RV and absent trabeculation portion, B) ventricular septal shunt (arrow), C) large atrial septal defect (lower arrows) and ventricular septal defect in Septum (upper arrow)

A 22-year-old woman was admitted to our clinic with mild dyspnea. The patient had a history of cardiac disease in her bother, who had died. Additionally, she had an abortion due to cardiac abnormalities in the fetus. Physical examinations revealed cyanosis (oxygen saturation at room air = 85%), clubbing, and systolic murmurs in the mitral area (+2/6+) radiating to the anterior axillary line. Two-dimensional Doppler echocardiography demonstrated the enlargement of the right atrium and the normal size of tricuspid valve annulus; however, in the subcostal view, there was hypoplasia of the apical portion of the right ventricle while the sub-pulmonary outflow was normal. The systolic pulmonary artery pressure was 30 mm Hg. A redundant interatrial septum with a large atrial septal defect and a bidirectional shunt was illustrated, and there was also evidence of a small apical muscular ventricular septal defect with no significant left-to-right shunting (Figure 1B & C).

**Figure 2:**
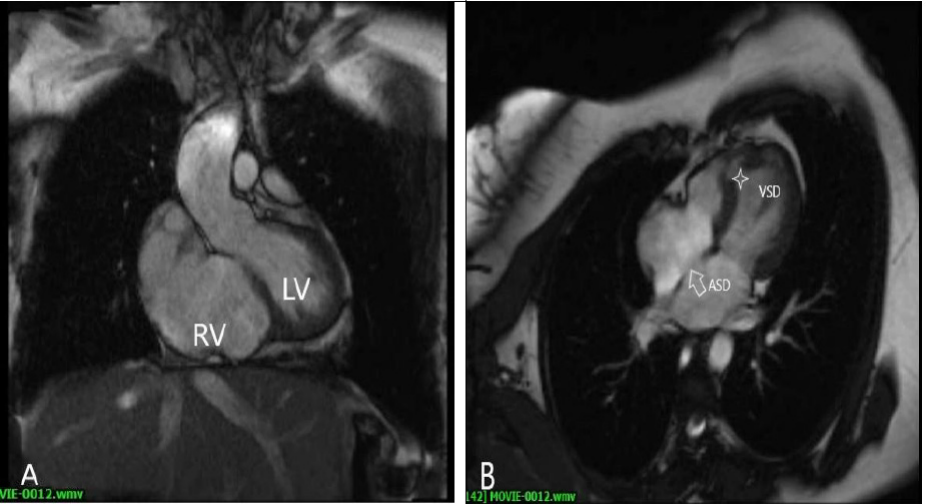
Cardiac magnetic resonance cine imaging confirmed: A) hypo-plastic RV with absent of trabeculation, B) small atrial septal defect and ventricular septal defect (asterisks)

Cardiac magnetic resonance revealed a normal volume and function for the left ventricle, while a large atrial septal defect and a small ventricular defect were seen along with a localized interventricular bulging of the septum at the site of the ventricular septal defect (Figure 2B). According to the cardiac magnetic resonance results, the right ventricle volume was in the lower normal limit with a mildly reduced function (Figure 2A).

Cardiac catheterization was performed in order to delineate the right ventricle and pulmonary artery hemodynamics and pressures. The data obtained from the right ventricle angiogram showed a small right ventricle with apical hypoplasia. The pressure of the right atrium and right ventricular end-diastolic pressure was increased, and evidence of an atrial septal defect with right-to-left shunting was observed. The saturation and pressure data from right-heart catheterization and cardiac magnetic resonance results are depicted in Table 1 and Table 2, respectively. Medical treatment with diuretics was done and subsequently, the patient was discharged. Re-evaluation was performed after 6 months by catheterization. The right ventricular end diastolic pressure and right atrium pressure decreased significantly, and the sizing balloon occlusion test showed no dramatic changes in the pressures. Accordingly, the atrial septal defect was closed with an Occlutech^®^ device (21 mm) percutaneously. After 1 month, the patient had no symptoms and cyanosis and follow-up after 2 years showed no symptoms.

## Case 2

A 36-year-old woman with a history of surgical atrial septal defect closure many years previously was referred to our hospital for an evaluation of her dyspnea and cyanosis. The patient mentioned a history of pulmonary stenosis in her son, who had undergone pulmonary valvuloplasty. Her previous surgical records at the time of surgery and after atrial septal defect closure showed that the surgeon had decided to open the patch because of her unstable hemodynamic status and the infeasibility of weaning from the cardiorespiratory pump.

Electrocardiogram (ECG) data showed sinus rhythms and incomplete right bundle branch block. Echocardiographic assessments revealed the normal size and mild dysfunction of the left ventricle, abnormalities in the apical part of right ventricle with moderate tricuspid regurgitation, and the normal size of the tricuspid valve annulus. In the interatrial septum, there were 2 residual septal defects, about 12 mm in size, with right-to-left shunting. The function and size of the pulmonary valve were normal. For further evaluations, cardiac catheterization was done and the right ventricle angiogram confirmed the hypo-plastic right ventricle at the apical portion with moderate systolic dysfunction. Additionally, cardiac pressure assessments showed elevated right atrium and ventricle pressures (Table 1 and Table 2). Cardiac magnetic resonance also showed the abnormal shape of the right ventricle with the right ventricle end-diastolic volume measurement at the lower normal limit. Given the severe symptoms in this case, the one and half ventricle repair (Glenn shunt, tricuspid valve repair and decreasing the ASD size) surgery was performed for her. Early postoperative assessments showed the improvement of function class and cyanosis.

**Table 1:** Pressure and saturation data of the patients from right heart catheterization

**Case**	**Pressure (mm Hg)**					**O**_2_ **saturation (%)**	
	**Left ventricle**	**Right ventricle**	**Right atrium**	**Pulmonary artery**	**Wedge**	**AO SO**_2_	**PV SO**_2_
1	120/0-10	33/0-17	17	33/17	17	85	97
2	120/0-10	35/0-18	18	35/18	18	86	97
3	120/0-8	31/0-20	20	30/10	10	78	98
4	120/0-12	26/0-12	12	26/12	12	82	95
5	100/0-10	25/0-11	11	20/11	11	79	98
6	110/0-10	20/0-10	10	20/10	10	88	94.5

AO, Aorta; PV, Pulmonary vein.

**Table 2:** Data of the analysis of the right and left heart chambers in cardiac magnetic resonance imaging

	**RV-EF%**	** RVEDVI (cc/m^2^) **	** RVESVI (cc/m^2^) **	**LVEF%**	** LVEDVI (cc/m^2^) **	** LVESVI (cc/m^2^) **
1	45	38	20	45	80	45
2	35	45	30	55	78	36
3	36	41	23	53	83	40
4	30	39	27	48	75	36
5	42	40	23	56	73	29
6	41	45	27	55	70	31

RV, Right ventricle; LV, Left ventricle; EF, Ejection fraction; EDVI, End-diastolic volume index; ESVI, End-systolic volume index.

## Case 3

A 35-year-old woman was admitted to our hospital with progressive dyspnea and cyanosis with oxygen saturation of 78% at room temperature. This patient had a family history of severe pulmonary stenosis in her child. Physical examinations revealed cyanosis and digital clubbing, and heart auscultation was normal. Echocardiographic and cardiac magnetic resonance assessments showed the normal size and function of the left ventricle; however, the right ventricle was small with a large patent foramen ovale leading to a right-to-left shunt.

Hemodynamic and cardiac pressure evaluations were done through catheterization (Table 1). The results showed increased right ventricle end diastolic pressure and right atrium pressure with evidence of right-to-left shunting. Given the patient’s cyanosis and the abovementioned symptoms, the Glenn procedure was performed. Further clinical evaluations of the patient soon after the surgical operation showed improvements in dyspnea and an increase in oxygen saturation (92%). Nonetheless, in further follow-ups, she complained of the exacerbation of her symptoms relative to the early days following surgery.

## Case 4

**Figure 3:**
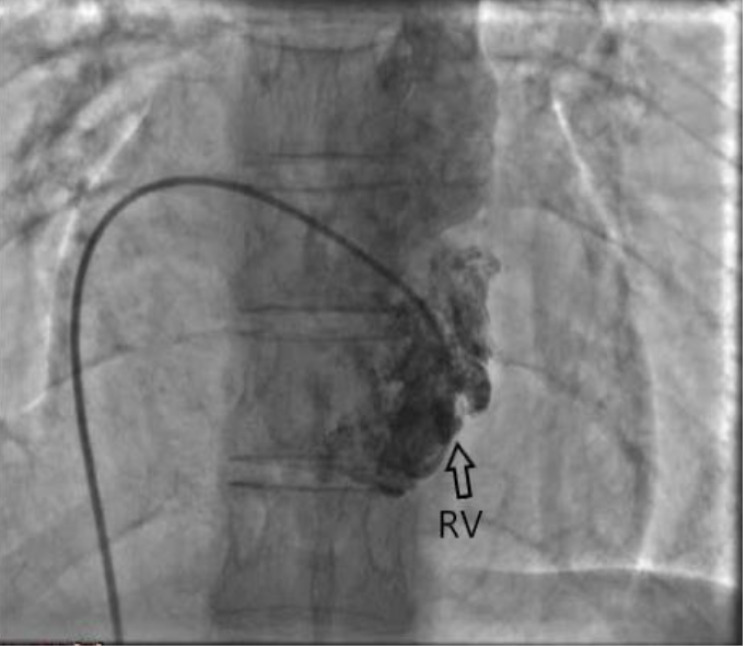
Right ventriculography in anterior-posterior view showed small and hypo-plastic right ventricle (arrows)

A 19-year-old man was referred to our clinic with the chief complaint of dyspnea. Physical examinations revealed cyanosis and clubbing with oxygen saturation of 82% at room temperature. ECG showed sinus rhythms without significant ST-T changes. Based on the echocardiographic data, the size and function of the left ventricle were normal, while the right ventricle was small with apical hypoplasia and moderate dysfunction. Assessments of the tricuspid valve revealed moderate tricuspid regurgitation and normal pulmonary artery pressure. There was also a large atrial septal defect with a bidirectional shunt. Angiographic and catheterization data showed increased right atrium pressure and right ventricle end diastolic pressure (Figure 3). On the basis of the information gathered, the patient underwent the Glenn surgery and the incomplete closure of the atrial septal defect. The surgery conferred significant alleviation in the patient’s symptoms.

## Case 5

**Figure 4:**
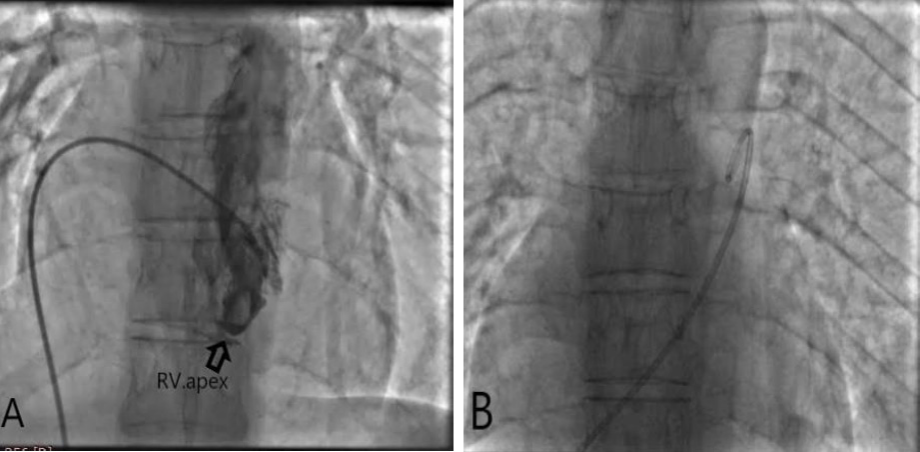
A) hypoplasia of right ventricle apex(arrows), B) coarse of right heart catheter from atrial septal defect

A 28-year-old man was admitted to the emergency service in our hospital with progressive dyspnea and cyanosis. Clinical examinations showed normal lungs, systolic murmurs, and oxygen saturation of 79% at room temperature. Echocardiographic evaluations showed a normal left ventricle, whereas there was hypoplasia in the apical part of the right ventricle with moderate dysfunction, moderate tricuspid regurgitation, and a moderately sized atrial septal defect with a right-to-left shunt. Cardiac catheterization confirmed elevated right chamber pressures. The right ventricle angiogram provided evidence of a hypoplastic right ventricle with moderate dysfunction, the absence of the apical part of the trabeculated right ventricle, and an atrial septal defect with right-to-left shunting. (Figure 4A& B). Given the patient’s severe symptoms and cyanosis, one and half ventricle repair (Glenn and incomplete closure of atrial septal defect) was suggested. The patient refused surgery, and medical treatment with spironolactone, digoxin, and furosemide was chosen for him.

## Case 6

A 20-year-old woman with a history of mild dyspnea and cyanosis from infancy referred to our hospital. She had a family history of congenital heart disease in her sister. Clinical examinations, followed by echocardiographic assessments, showed a normal left ventricle, a small right ventricle (Figure 1A) with moderate dysfunction, and a large stretched patent foramen ovale with right-to-left shunting.

Cardiac magnetic resonance confirmed all the above mentioned results. Further cardiac catheterization illustrated the absence of the apical part of the right ventricle, increased right atrium pressure and right ventricle end diastolic pressure and evidence of a patent foramen ovale with bidirectional shunting. Medical treatment with diuretics was started for the patient; and 3 months after the medical therapy, catheterization and the sizing balloon occlusion test were performed. Given the absence of any dramatic changes in right atrium pressure and right ventricle end diastolic pressure, the patent foramen ovale was closed with an atrial septal defect Occlutech^®^ device (18 mm). Post procedurally, the patient’s cyanosis was eliminated and she was asymptomatic in her follow-ups.

## Discussion

Isolated right ventricle hypoplasia is a rare anomaly that in which the trabecular components of the right ventricle were absent or less developed, without significant tricuspid or pulmonary valve malformations. From 1950 to 2009, there were 74 reports of patients suffering from isolated right ventricle hypoplasia in 41 different studies as reviewed by Lombardi et al.^1^ In the present study, we summarized data from 2010 to 2019 (Table 3).

**Table 3:** Previous studies on patients with isolated right ventricular hypoplasia (from 2009 to 2019)

**Year**	**First author (Ref)**	**Age/sex**	**ASD/PFO**	**Symptoms**	**Diagnostic test**	**Therapy**	**TV, RV outflow**
2012	Kim^20^	14 years/F	ASD	Cyanosis	Echo and MRI	One and a half ventricle repair and tricuspid annuloplasty	Small tricuspid annulus
2012	Kim^20^	6 years/F	ASD	Cyanosis and exertional dyspnea	Echo and MRI	One and half ventricle repair	Small tricuspid annulus
2016	Mohan^21^	26 years/F	ASD	Clubbing and cyanosis	Echo	Anti-failure therapy followed by repeated phlebotomy	Absent pulmonary valve and bicuspid aortic valve
2016	Zhu^22^	45 years/M	N/M	Chest congestion	Echo and angiography	RCA closing	Normal tricuspid and pulmonary valves
2016	Reddin^23^	58 years/F	ASD	Fatigue and right-sided heart failure	Echo, MRI, and cardiac catheterization	TV repair and right reduction atrioplasty	Abnormal pulmonary valve cusp mobility and malcoaptation of TV leaflets
2016	Lombardi^1^	Newborn/F	ASD	Cyanosis	Echo	Oxygen administration	Normal tricuspid and pulmonary valves
2016	Lombardi^1^	36 weeks/F	ASD	Cyanosis	Echo and angiography	Surgery, dobutamine administration, and mechanical ventilation	Small tricuspid valve and normal pulmonary valve
2016	Lombardi^1^	Newborn/M	ASD	Cyanosis	Echo	Supplemental oxygen	Normal tricuspid valve and dysplastic pulmonary valve
2017	Zhou^3^	62 years/M	PFO	Abdominal distention, nausea, and lower-extremity edema	Echo and CT angiography	Medical follow up	Normal tricuspid and pulmonary valves
2018	Rao^4^	10 months/M	ASD	Cyanosis	Echo and MRI	Bidirectional Glenn procedure and ASD closure	Minimal obstruction across the tricuspid inflow, the TV was borderline with doming and dysplasia
2019	Qasim^24^	2 patients/ 23 years/ M&F	PFO	Asymptomatic	Echo and MRI	None	Normal

M, Male; F, Female; ASD, Atrial septal defect; PFO, Patent foramen ovale; Echo, Echocardiography; MRI, Magnetic resonance imaging; TV, Thoracic valve; RV, Right ventricle; N/M, not mentioned; CT, Computerized tomography; RCA, Right coronary artery

Only a few cases of right ventricle hypoplasia occur in adulthood, and most of them tend to manifest themselves in infancy.

### Pathophysiology

The right ventricle consists of 3 parts: the atrioventricular valve as an inflow tract, the trabecular portion, and the outflow tract.^5^ Hypo-plastic right ventricle is characterized when 1 or more of these 3 components have an anomaly and cause a reduction in the chamber size. Hypo-plastic right ventricle could be associated with different abnormalities such as pulmonary valve atresia and tricuspid valve atresia as well as other congenital defects such as interventricular septal defects.^6^ The underdevelopment of the trabecular portion but normally developed pulmonary and tricuspid valves could lead to a reduction in the right ventricle size, characterized as isolated right ventricle hypoplasia, which is a rare disease with only a few cases reported.^7^ The main cause of isolated right ventricle hypoplasia in most cases is unknown; in some cases, however, the cause appears to be familial.^8,9^ We detected this association in our cases 1, 2, 3, and 6; however, our genetic tests on these cases aimed at finding a mutation or a single-nucleotide polymorphism yielded no evidence to confirm this hypothesis. The existing literature lacks a reliable estimation as regards predominance between sexes in isolated right ventricle hypoplasia.^1^

### Clinical presentation

As a mentioned earlier, isolated right ventricular hypoplasia is a cyanotic congenital heart disease without significant associated anomaly that clinical manifestations of this disease and the onset of symptoms are highly variable depending on the severity of hypoplasia. In cases with less malformation, symptoms such as cyanosis, dyspnea, and digital clubbing may be found later; whereas in more severe cases, congestive heart failure and cyanosis can appear during infancy.^10^ In general, the severity and timing of the onset of symptoms vary greatly from patient to patient, depending on the severity of the underlying abnormality and degree of right ventricle hypoplasia.^11^ In addition, published articles have reported few cases of disease diagnosis during surgery.^12^

### Diagnostic methods

Complementary diagnostic tests are crucial to the differential diagnosis of isolated RVH from other disease with cyanosis, and it is often determined by echocardiography, CMR, or hemodynamic assessments.

A simplest test is EKG that is of importance, and maybe showed decreased RV electrical forces and signs of right atrial or biatrial hypertrophy, cardiac axis deviation to the left, and sometimes atrioventricular conduction disorders, but in many patients, it is not a diagnostic modality.^13,14^

Chest X-ray contributes little to the diagnosis, as it may show normal cardiac silhouette, cardiomegaly and/or normal or decreased pulmonary blood flow.^15^ Echocardiography is another diagnostic method that is simple and available. We used it as an earlier modality for evaluation of these patients and usually shows a decreased right ventricle size and the hypoplasia of the trabecular portion with no anomalies in the tricuspid and pulmonary valves. Besides, the patent foramen ovale or interatrial septal defect appear in most patients as compensatory components with right to left shunt.^16^ Cardiac magnetic resonance can provide comprehensive evaluations, and it is widely used in the assessment of congenital heart defects. It does not use ionizing radiation or potentially nephrotoxic means of contrast and provides more comprehensive cardiac evaluation. Cardiac magnetic resonance provides precise data of the ventricular function and volume and the accurate quantification of right-to-left shunts via atrial septal defect or patent foramen ovale.^17^

Cardiac catheterization demonstrates a rise in end-diastolic pressures and also in right atrial pressure. The presence of a mixed shunt at the atrial level in the absence of pulmonary hypertension, tricuspid or pulmonic valvular disease demonstrates the restriction to inflow caused by the hypo-plastic right ventricle.^18^ Furthermore, an increase in the right atrial pressure and in initial and final diastolic pressures demonstrates reduced ventricular filling capacity. Moreover, right-left or bidirectional shunts can be found, as well as an oxygen saturation of 66% to 90%.^11^ One of the best modalities for evaluating the right ventricle shape and especially detecting the absence of the right ventricle apical part in right ventricle angiography.

A precise evaluation of isolated right ventricle hypoplasia needs multimodality imaging.

### Management

In patients with isolated right ventricle hypoplasia, decision-making vis-à-vis medical treatment, catheter intervention, or surgical treatment depends on the severity of the disease and symptoms.

The right ventricular size measurement is the most important factor in choosing appropriate approach. Sizing balloon occlusion test in Cath lab or intraoperative period can provide informative data to identify eligible patient for simple closure of ASD by device or surgical approach. Atrial septal defect should be closed only when the right ventricle can adapt to the increased volume load.^19^

In the cases with severe hypoplasia whom had significant elevation in RA pressure and RVEDP after balloon occlusion test, incomplete closure of ASD with SVC to pulmonary artery connection (Glenn shunt) recommended. The aims of the surgical repair of isolated right ventricle hypoplasia in this situation, are to offload the small right ventricle through a direct connection between the vena cava and the pulmonary artery and to completely or incompletely close the atrial septal defect to improve cyanosis. Sometimes in cases with very severe hypoplasia Fontan operation is the better surgical approach. Our postoperative evaluations of the patients showed that all the chosen interventions were successful in alleviating the symptoms.

## Conclusion

With regard to the different presentation of isolated right ventricle hypoplasia, we suggest meticulous examinations and evaluations of patients with atrial septal defect presenting with cyanosis. The evaluations of our cases revealed the importance of a combination of echocardiography, angiography, and cardiac magnetic resonance along with physical examinations and clinical history taking when it comes to a precise assessment of patients and decision-making about medical or surgical treatment. The early interventions for patients with isolated right ventricle hypoplasia with decreased pulmonary blood flow and cyanosis may include atrial septal defect catheter intervention or surgical closure when the right atrium pressure and right ventricular end diastolic pressure is reasonable (especially good hemodynamic response after temporary balloon occlusion) or Glenn and or Fontan surgery for offloading the RV and in some patients one and a half ventricle repair.

## Competing interests

None declared.

## Ethical approval

Ethical approval was waived given the retrospective observational design of the study. We obtained each patient consent to anonymously publish their case.

## Acknowledgements

We thanks our kind patients and our respectful colleagues who made this report possible.

## Funding

None.
